# Effect of Early Supplemental Formula Intervention on Body Weight and Hyperbilirubinemia in Neonates During 72 h After Birth

**DOI:** 10.3389/fped.2021.625536

**Published:** 2021-05-26

**Authors:** Lu-Lu Zhao, En-Pei Lee, Raymond Nein-Chen Kuo, Stephen Shei-Dei Yang, Su-Cheng Huang, Han-Ping Wu

**Affiliations:** ^1^Department of Pediatrics, Taipei Tzu Chi Hospital, New Taipei, Taiwan; ^2^Department of Medicine, School of Medicine, Tzu Chi University, Hualien, Taiwan; ^3^Division of Pediatric Critical Care Medicine, and Pediatric Sepsis Study Group, Department of Pediatrics, Chang Gung Memorial Hospital at Linko, Taoyuan, Taiwan; ^4^College of Medicine, Chang Gung University, Taoyuan, Taiwan; ^5^Institute of Health Policy and Management, College of Public Health, National Taiwan University, Taipei, Taiwan; ^6^Innovation and Policy Center for Population Health and Sustainable Environment, College of Public Health, National Taiwan University, Taipei, Taiwan; ^7^Division of Urology, Taipei Tzu Chi Hospital, New Taipei, Taiwan; ^8^Division of Obstetrics and Gynecology, Taipei Tzu Chi Hospital, New Taipei, Taiwan; ^9^Department of Pediatric Emergency Medicine, China Medical University Children's Hospital, Taichung, Taiwan; ^10^Department of Medicine, School of Medicine, China Medical University, Taichung, Taiwan; ^11^Department of Medical Research, China Medical University Children's Hospital, China Medical University, Taichung, Taiwan

**Keywords:** dehydration, jaundice, breastfeeding, body weight loss, neonate

## Abstract

**Background:** The full breastfeeding may lead to insufficient milk intake of newborns and increase the rate of body weight loss (BWL). Severe BWL was generally believed as a cause of significant hyperbilirubinemia in newborn babies. The study aimed to investigate the effect if early supplemental feeding in newborns with birth weight loss at the first 3 days after birth could decrease the rate of hyperbilirubinemia 72 h of birth.

**Methods:** A total of 395 neonates with gestational age >37 weeks and birth body weight >2500g were prospectively collected between 2016 and 2018. We analyzed 280 neonates with BWL rate reaching the predictive value (4.5%, 7.5%, and 8% on the first, second, third day after birth, respectively) for subsequent hyperbilirubinemia after 72 hours after birth. The enrolled cases were divided into four subgroups as interventional consecutive milk supplement for 0, 1, 2, and 3 days after birth for further analysis

**Results:** For newborns with BWL reaching the predictive value on the first day after birth, the serum bilirubin levels were lower in the experimental group than those in the non-involved control group (*p* < 0.05). For newborns with three consecutive days of interventional milk supplementation, the serum bilirubin levels at the 72 h after birth showed the lowest levels compared with the other sub-groups with two consecutive days and one consecutive day of interventional milk supplementation (*p* < 0.05). Moreover, there was a significantly decreasing trend in the consecutive days of interventional milk supplementation (*p* < 0.05).

**Conclusion:** Newborns with BWL over 4.5% on the first day after birth receiving early intervention milk supplementation could significantly reduce serum bilirubin levels at the

72 h after birth. The more days of consecutive milk supplementation after birth may lead to the lower the 72 h serum bilirubin levels. It is recommended to early and consecutive milk supplementation after birth to be an effective way in reducing serum bilirubin levels.

## Introduction

The Baby-Friendly Hospital Initiative (BFHI) is a global effort to promote breastfeeding launched in 1991 and was first supported in Taiwan in 2000. Hospitals across Taiwan urge exclusive breastfeeding to become maternal- and child-friendly hospitals and continue to promote the BFHI standards. However, new mothers may lack breastfeeding skills to increase milk volume, which often leads to clinically insufficient milk intake in newborns. This can result in weight loss and increased hospitalization rates due to hyperbilirubinemia (1–9).

Following implementation of the BFHI program, the breastfeeding rate at a Taiwan medical center increased from 92.18 to 97.15%. A corresponding increase in cases of hyperbilirubinemia (10.88 to 23.77%) was also observed due to weight loss and improper breastfeeding (2). Risk factors for neonatal hyperbilirubinemia include polycythemia, neonatal hemolytic disease, glucose-6-phosphate dehydrogenase deficiency, and exclusive breastfeeding (1, 10). Interventional formula supplementation, particularly in the first 3 days after birth, can prevent hyperbilirubinemia due to inadequate breastfeeding and dehydration (1, 3, 7).

However, there is no accepted body weight loss (BWL) rate to predict persistent hyperbilirubinemia in clinical practice. Moreover, research indicating the need for formula supplementation to prevent severe hyperbilirubinemia remains equivocal. Use of supplemental formula in newborns has been suggested at >7% BWL after 3 days of birth (11–14), whereas the Academy of Breastfeeding Medicine advises supplementation at 8 to 10% BWL accompanied by delayed lactogenesis on day 5 or later (15). The current study conducted a prospective clinical survey to investigate whether early supplemental feeding in newborns that reached retrospective predictive BWL rates within the first 3 days after birth could decrease hyperbilirubinemia rates.

## Methods

### Study Population

This prospective interventional study occurred between 2016 and 2018. Neonates born after at least 37 gestational weeks with a birth body weight (BW) >2,500 g were identified at a medical hospital. Of the 395 identified neonates, 115 were excluded from the study, leaving 280 enrolled for analysis. The exclusion criteria were early onset hyperbilirubinemia (within 48 h), pathological neonatal jaundice (including hemolysis, glucose-6-phosphate dehydrogenase deficiency, congenital infections, and congenital hypothyroidism), birth injury, prenatal asphyxia, major organ anomalies, cephalohematoma, maternal or infant exposure to unapproved drugs, maternal gestational diabetes, and infants exclusively fed formula. The study was approved by the Institutional Review Board of Taipei Tzu Chi Hospital (registration number: 04-X34-088 and the date of registration as 08/06/2016). All methods were performed in accordance with the relevant guidelines and regulations. Informed consent was obtained from a legal guardian for study participation.

### Procedure

**Figure 1:**
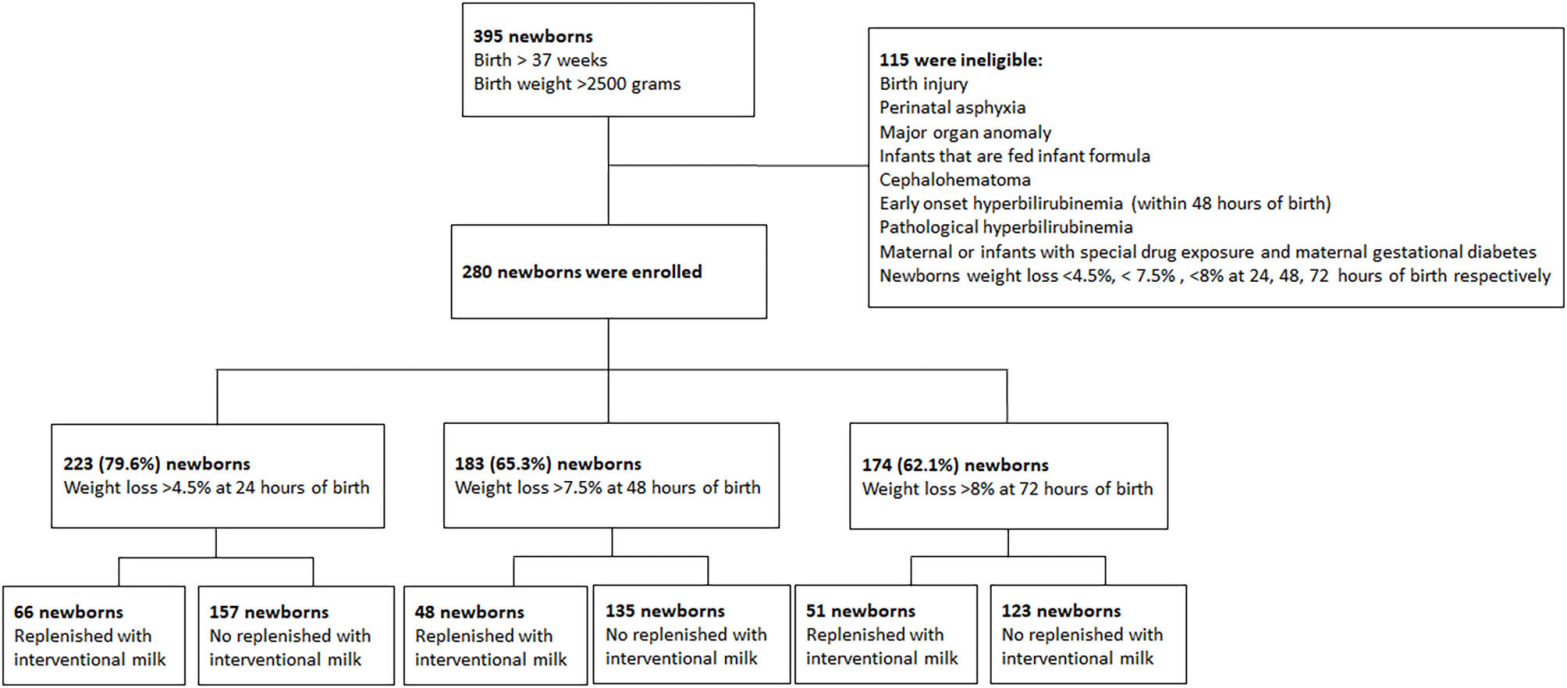
Flow chart of included patients.

The 280 eligible neonates were identified as falling into 4 subgroups based on breastfeeding status (Figure 1):

Group I (control group): Formula supplementation for 0 days (*n* = 164)

Group II: Formula supplementation for 1 consecutive day (*n* = 18)

Group III: Formula supplementation for 2 consecutive days (*n* = 32)

Group IV: Formula supplementation for 3 consecutive days (*n* = 66)

They were further broken down into categories based on percentage of BWL at each time point: >4.5% at 24 h, >7.5% at 48 h, and >8.0% at 72 h after birth. Neonates were supplemented with 10 ml/kg interventional formula per meal.

The following variables were collected: gender, gestational age, birth BW, BW 24 h after birth (first day), BW 48 h after birth (second day), BW 72 h after birth (third day), delivery methods (normal spontaneous delivery or cesarean section), feeding method (exclusive breastfeeding or breastfeeding combined with formula), BWL rate during the 3 days after birth, daily number of urinations, the daily weight change obtained from the neonatal inpatient record, and bilirubin levels at 72 h after birth. Serum microbilirubin was routinely checked in infants by heel stick for blood sampling using direct spectrophotometric methods at 3 and 7 days after birth. Bilirubin levels were measured using an APEL Model: BR-501 bilirubin meter. Neonates were weighed daily for at least 3, and up to 5, days after birth. Measurements were compared with the first day to identify rates of dehydration and BWL. Gestational age was not analyzed because most infants were born at 39–40 gestational weeks.

### Statistical Analysis

All statistical analyses were performed using Kruskal–Wallis tests, Jonckheere–Terpstra tests, and multiple linear regression analysis. Bilirubin values were compared across subgroups. Correlations between BWL rate within 72 h and the total bilirubin level 72 h after birth were analyzed. Correlations between the TSB level and related clinical parameters were also analyzed. Multiple linear regression was used to analyze hematocrit levels across the neonates based on days of interventional formula supplementation. The results are reported as percentages and mean ± standard deviation. The differences between groups are presented as 95% confidence intervals. Probability levels of <0.05 were considered significant. Statistical analyses were performed using SPSS software (version 22.0, SPSS Inc., Chicago, IL, USA).

## Results

**Figure 2:**
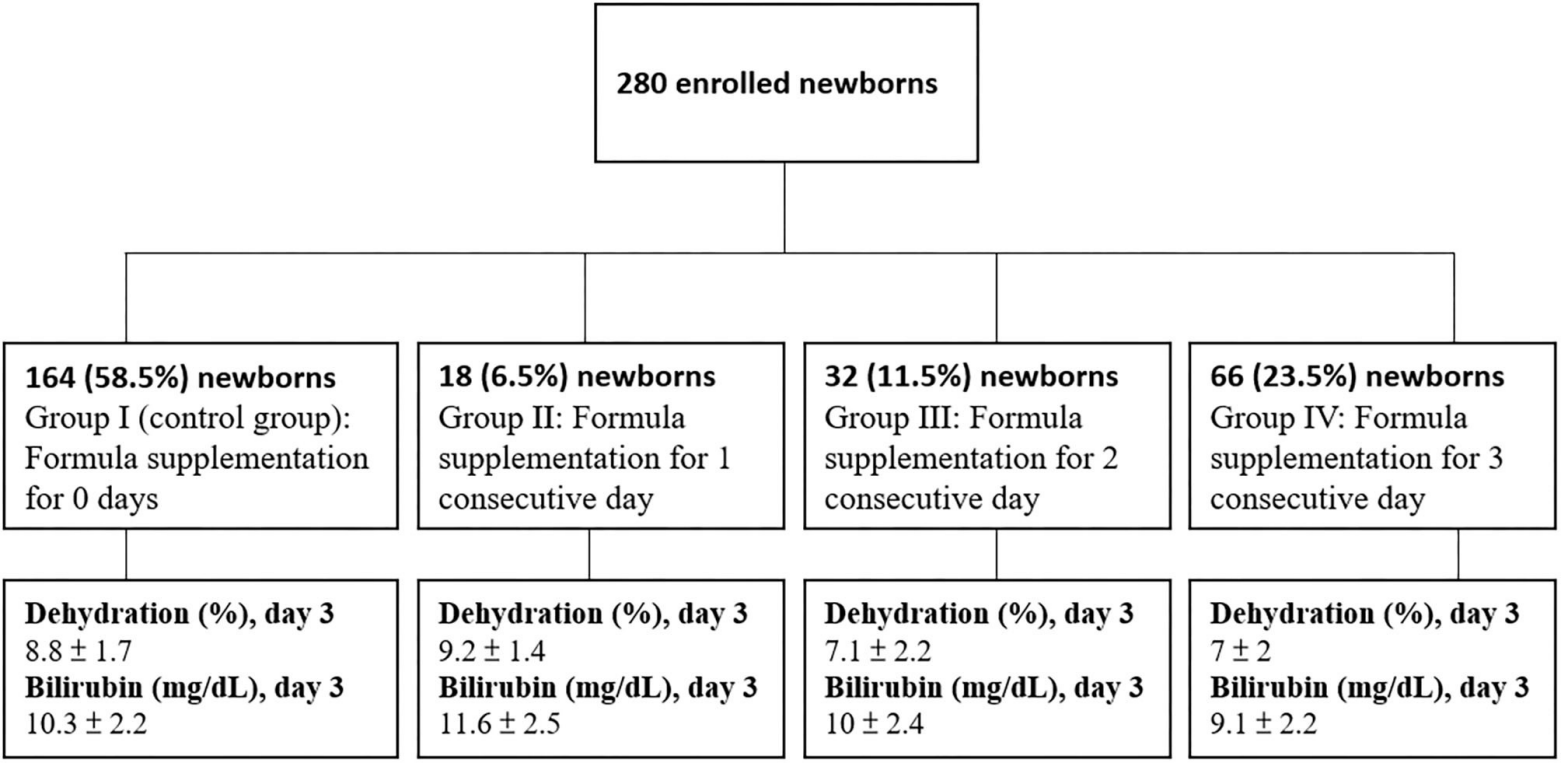
Flow chart of 4 subgroups based on formula supplementation status.

The mean gestational age was 38.6 weeks and mean birth BW was 3,136.1 grams for the 280 neonates included in the analyses (Table 1). Mean percent of BWL was 5.6% at 24 h, 7.9% at 48 h, and 8.2% at 72 h. Comparisons between the number of interventional formula days are shown in Table 2. There was no significant difference in percentage of BWL between interventional formula supplementation groups on day 1. However, there was a significant difference in percentage of BWL between interventional formula supplementation groups on days 2 and 3 (*p* < 0.001). There was a decreasing trend in percentage of BWL for day 2 and day 3. The percentage of BWL percentage decreased as the days of consecutive supplemental formula increased (Figure 2). In addition, day 3 serum bilirubin levels were significantly lower in newborns with 3 consecutive days of supplemental formula compared with 1 and 2 consecutive days of supplemental formula (*p* < 0.05) (Figure 2). There were no significant differences in percentage of males, birth BW, HCT, or day 7 bilirubin levels between number of interventional formula days.

**Table 1:** Newborn demographic data.

**Variables**	**All newborns (*n* = 280)**	**Day 1 (*n* = 280)**	**Day 2 (*n* = 280)**	**Day 3 (*n* = 280)**
GA (mean ± SD)	38.7 ± 0.9			
Types of delivery				
C/S, n (%)	59 (21.3)			
NSD, *n* (%)	218 (78.7)			
Sex (male), *n* (%)	122 (43.6)			
Parity, *n* (%)				
P:0	87 (31.1)			
P:1	106 (37.9)			
P:2	51 (18.2)			
P:3	32 (11.3)			
P:4	3 (1.1)			
P:5	1 (0.4)			
Birth BW (g) (mean ± SD)	3136.1 ± 269.4			
BW (g) (mean ± SD)		2962.1 ± 264.1	2886.9 ± 250.4	2879.8 ± 248.3
Dehydration (%) (mean ± SD)		5.6 ± 1.6	7.9 ± 1.7	8.2 ± 2
Newborns receiving interventional milk, *n* (%)		74 (26.4)	99 (35.3)	107 (38.2)

*GA, gestational age; NSD, normal spontaneous delivery; BW, body weight*.

**Table 2:** Comparisons between newborns with and without interventional formula supplementation.

**Variables**	**No interventional milk (*N* = 164)**	**Interventional milk (*****N*** **=** **116)**				
		**Last 1 day (*N* = 18)**	**Last 2 days (*N* = 32)**	**Last 3 days (*N* = 66)**	***P*-value^**a**^**	***P*-value^**b**^**
GA (mean ± SD)	38.8 ± 0.9	38.5 ± 0.8	38.3 ± 0.9	38.6 ± 1	0.037	0.023
Types of delivery, *n* (%)					
C/S	15 (9.3)	6 (33.3)	9 (28.1)	29 (43.9)	<0.001	<0.001
NSD	146 (90.7)	12 (66.7)	23 (71.9)	37 (56.1)		
Sex (male), *n* (%)	77 (47)	7 (38.9)	11 (34.4)	27 (40.09)	0.529	0.260
Birth BW (g) (mean ± SD)	3144.6 ± 264.7	3184.4 ± 271.3	3152.7 ± 276.5	3093.6 ± 278	0.453	0.226
BW (g), day 1 (mean ± SD)	2973.1 ± 257.8	2993.1 ± 276.1	2978.4 ± 272.4	2918.2 ± 273.1	0.405	0.164
Dehydration (%), day 1 (mean ± SD)	5.5 ± 1.7	6 ± 2.2	5.5 ± 1.4	5.7 ± 1.3	0.331	0.231
BW (g), day 2 (mean ± SD)	2885.6 ± 246.7	2903.6 ± 255.8	2910.2 ± 258.2	2874.2 ± 258.7	0.938	0.789
Dehydration (%), day 2 (mean ± SD)	8.2 ± 1.4	8.8 ± 1.3	7.7 ± 2.2	7.1 ± 1.7	<0.001	<0.001
BW (g), day 3 (mean ± SD)	2868.9 ± 245.9	2890 ± 236.7	2944.7 ± 263.6	2874.5 ± 251.4	0.620	0.616
Dehydration (%), day 3 (mean ± SD)	8.8 ± 1.7	9.2 ± 1.4	7.1 ± 2.2	7 ± 2	<0.001	<0.001
HCT, day 1 (mean ± SD)	46.2 ± 4.4	45.5 ± 4.2	46.5 ± 4.3	46 ± 3.9	0.922	0.654
Bilirubin (mg/dL) (mean ± SD) (day 3)	10.3 ± 2.2	11.6 ± 2.5	10 ± 2.4	9.1 ± 2.2	<0.001	<0.001
Bilirubin (mg/dL) (mean ± SD) (day 7)	12.2 ± 2.7	11 ± 2.6	11.5 ± 2.8	11.3 ± 3.5	0.513	0.193

*GA, gestational age; NSD, normal spontaneous delivery; BW, body weight; HCT, hematocrit*.

*P-value (a) by Kruskal–Wallis test (trend and gradually increase)*.

*P-value (b) by Jonckheere–Terpstra Test*.

Comparison of supplemental formula vs. no intervention based on percentage of BWL at different timepoints are shown in Table 3 (>4.5% at 24 h), Table 4 (>7.5% at 48 h), and Table 5 (>8% at 72 h). Early intervention with supplemental formula at 24 h in infants with >4.5% BWL significantly reduced day 3 serum bilirubin levels and percentage of BWL (Table 3). The effect of formula intervention was not present in neonates with >7.5% BWL at 48 h or >8.0% BWL at 72 h. There were no effects of formula intervention on birth BW, HCT, or day 7 bilirubin based on percentage of BWL at any timepoint.

**Table 3:** Comparisons between no formula and interventional formula in newborns with BWL >4.5% at 24 h.

	**Weight loss** **>4.5% at 24 h of birth (*****N*** **=** **223)**		
**Variables**	**Interventional milk (*n* = 66)**	**No interventional milk (*n* = 157)**	***P*-value**
GA (mean ± SD)	38.5 ± 1.1	38.7 ± 0.9	0.173
Types of delivery			
C/S, *n* (%)	31 (46.9)	21 (13.4)	<0.001^*^
NSD, *n* (%)	35 (53.1)	135 (86.6)	
Sex (male), *n* (%)	28 (42.4)	67 (42.7)	0.061
Birth BW (g) (mean ± SD)	3083 ± 270.4	3115 ± 262.5	0.168
BW (g) (mean ± SD)	2902.8 ± 261.4	2915 ± 249.8	0.296
Dehydration (%) (mean ± SD)	6 ± 0.9	6.1 ± 1.2	0.04
HCT (mean ± SD)	45.5 ± 3.9	45.8 ± 4.4	0.356
Bilirubin (mg/dL) (mean ± SD) (day 3)	9.1 ± 2.2	10 ± 2.9	<0.001^*^
Bilirubin (mg/dL) (mean ± SD) (day 7)	11.6 ± 2.7	10.9 ± 3.5	0.212

*GA, gestational age; NSD, normal spontaneous delivery; BW, body weight; HCT, hematocrit*.

* *Statistical significance was set at p <0.05*.

**Table 4:** Comparisons between no formula and interventional formula in newborns with BWL >7.5% at 48 h.

	**Weight loss** **>7.5% at 48 h of birth (*****N*** **=** **183)**		
**Variables**	**Interventional milk (*n* = 48)**	**No interventional milk (*n* = 135)**	***P*-value**
GA (mean ± SD)	38.7 ± 0.9	38.5 ± 0.9	0.122
Types of delivery			
C/S, *n* (%)	14 (29.1)	16 (11.8)	0.006
NSD, *n* (%)	34 (70.9)	117 (88.2)	
Sex (male), *n* (%)	21 (43.8)	62 (45.9)	0.795
Birth BW (g) (mean ± SD)	3150.5 ± 306.7	3162.5 ± 278.9	0.803
BW (g) (mean ± SD)	2874.4 ± 287.5	2882.8 ± 261.1	0.852
Dehydration (%) (mean ± SD)	8.8 ± 1.1	8.9 ± 1.1	0.678
HCT (mean ± SD)	46.1 ± 4.1	46.2 ± 4.5	0.890
Bilirubin (mg/dL) (mean ± SD) (day 3)	10 ± 2.5	10.5 ± 2.4	0.223
Bilirubin (mg/dL) (mean ± SD) (day 7)	11.3 ± 3.4	11.9 ± 2.8	0.378

*GA, gestational age; NSD, normal spontaneous delivery; BW, body weight; HCT, hematocrit*.

**Table 5:** Comparisons between no formula and interventional formula in newborns with BWL >8% at 72 h.

	**Weight loss** **>8% at 72 h after birth (*****N*** **=** **174)**		
**Variables**	**Interventional milk (*n* = 51)**	**No interventional milk (*n* = 123)**	***P*-value**
GA (mean ± SD)	38.7 ± 0.9	38.7 ± 0.9	0.665
Types of delivery			
C/S, *n* (%)	19 (37.2)	14 (11.4)	<0.001^*^
NSD, *n* (%)	32 (62.8)	106 (88.6)	
Sex (male), *n* (%)	15 (29.4)	55 (44.7)	0.061
Birth BW (g) (mean ± SD)	3193.1 ± 261.1	3157.9 ± 253.4	0.410
BW (g) (mean ± SD)	2897.9 ± 236.8	2858 ± 238.5	0.318
Dehydration (%) (mean ± SD)	9.2 ± 0.9	9.5 ± 1.1	0.133
HCT (mean ± SD)	45.9 ± 3.8	46.3 ± 4.4	0.605
Bilirubin (mg/dL) (mean ± SD) (day 3)	10.4 ± 2.5	10.55 ± 2.3	0.620
Bilirubin (mg/dL) (mean ± SD) (day 7)	11.3 ± 3.9	12.3 ± 2.8	0.186

*GA, gestational age; NSD, normal spontaneous delivery; BW, body weight; HCT, hematocrit*.

* *Statistical significance was set at p <0.05*.

The multiple linear regression results for the effect of none, one, 2, or 3 consecutive days of supplemental formula on day 3 serum bilirubin levels are shown in Table 6. The analysis revealed that, although hematocrit did not differ based on formula intervention, it was a significant factor that may affect hyperbilirubinemia severity. Day 3 serum bilirubin levels in neonates with supplemental formula for 3 consecutive days were reduced by 1.009 mg/dL compared with the control group (*p* < 0.05). Thus, the occurrence of hyperbilirubinemia may be effectively reduced in 72 h.

**Table 6:** Multiple linear regression analysis for the effects of interventional formula supplementation on serum bilirubin levels.

**Variables**		**Estimate**	**SE**	***P*-value**
(Intercept)		2.566	1.476	0.082
Interventional milk	For 3 days	−1.009	0.327	0.002
	For 2 days	−0.317	0.427	0.457
	For 1 day	1.278	0.578	0.027
No interventional milk		0.000		
HCT, day 1		0.168	0.032	<0.001

*P-value by multiple linear regression*.

*HCT, hematocrit*.

## Discussion

Reported incidences of hyperbilirubinemia have increased following implementation of the BFHI program to promote breastfeeding. In Taiwan, incidences of hyperbilirubinemia at one northern Medical Center grew from 10.88 to 23.77% during a study conducted in the year 2000 (2). There is a correlation between BWL and hyperbilirubinemia in neonates in the first 3 days after birth (1–3). Based on this relationship, retrospective reports have suggested an ideal cutoff value for BWL rates over the 3 days post-birth to indicate necessity of supplemental formula diets to prevent hyperbilirubinemia (1, 3, 11). Early intervention with supplemental formula may reduce neonatal hyperbilirubinemia when neonate BWL is below the cutoff values.

According to the current findings, when BWL rate was >4.5% at 24 h, >7.5% at 48 h, and >8% at 72 h, supplemental formula may be indicated to reduce neonatal hyperbilirubinemia 3 days after birth. The results of the multiple linear regression analysis also showed that 3 consecutive days of supplemental formula significantly reduced day 3 serum bilirubin levels. This may indicate that early and continuous supplemental formula better prevents hyperbilirubinemia. However, 2 consecutive days of supplemental formula did not significantly reduce day 3 bilirubin levels. These differences may be due to the timing of supplemental formula intervention within the experimental groups. If a neonate did not meet BWL criteria until later in the study, such as showing >8% BWL at 72 h, then supplemental formula was only indicated the third day. Serum bilirubin levels were tested on the third day. Thus, supplemental formula given outside of the first 24 h after birth may not be early enough to significantly affect bilirubin levels.

Most medical institutions follow the Academy of Breastfeeding Medicine protocol, which indicates that breast milk should only be supplemented when newborn percentage of BWL is >10% (15). Therefore, exclusive feeding with breast milk is highly encouraged and recommended. However, use of supplemental formula may actually be indicated before percentage of BWL reaches 10%. The current guidelines and protocols may result in a critical loss of opportunity for supplemental formula intervention. This may result in dangerous and unnecessary severe dehydration in newborns, as well as an increase in neonatal hypernatremia and hyperbilirubinemia. Hyperbilirubinemia related conditions may arise, including the need for phototherapy, additional hospitalization costs, and additional hospital stays that result in family concern and inconvenience. Moreover, repeated hospitalization results in separation of the mother and child, which may increase the probability of maternal postpartum depression. Despite this, many medical institutions promote exclusive breastfeeding policies until BWL is >10%. According to the recommendations of maternal and child assessments, percentage of BWL in newborns must be >10% before supplementing breast milk with formula (15). If BWL is >7% 24 h after birth, the newborns would exceed the normal range of neonatal dehydration within the first 24 h (4.2–6.5%) (16), but still not meet criteria for supplemental formula.

Thus, it is essential to take other factors into account to avoid possible complications. The physiological model of neonatal physiological dehydration was developed in the United States in 2014 to identify early interventional timepoints for formula supplementation to avoid severe dehydration (16). However, this model may not be applicable in other areas, such as Asia, due to different ethnicities, cultures, and birth weights, which may lead to differences in the physiological dehydration ratio of newborns. Therefore, it is important to establish regionally independent BWL nomograms for newborns.

There are some limitations to this prospective study. Participants were enrolled from a single medical center, leading to a small sample size that may not be widely representative. In addition, the amount of breast milk fed to the newborns in both the control and experimental groups could not be determined. Under the BFHI rules, the mother's breast milk cannot be squeezed into a bottle to feed to the infants, and therefore could not be calculated or controlled in this study.

Moreover, there are also many factors determining successful initiation of breastfeeding including the previous experience of the mother, labor room practices (e.g., early essential newborn care), the motivation of mother, the maternal prenatal and postpartum sentiments (family support and encouragement, postpartum depression), etc. Although these factors may have a large impact, they were not assessed.

## Conclusion

Newborns with BWL >4.5% 24 h after birth showed significantly reduced day 3 serum bilirubin levels when breast milk was supplemented with formula. An increased number of consecutive days with supplemental formula after birth may further lower day 3 serum bilirubin levels. Early and continuous formula supplementation is recommended as an effective way to prevent neonatal hyperbilirubinemia 72 h after birth.

## Data Availability Statement

The original contributions presented in the study are included in the article/supplementary material, further inquiries can be directed to the corresponding author.

## Ethics Statement

The study protocol was approved by the Institution Review Board and ethics committee of Taipei Tzu Chi hospital on June 8, 2016. IRB No.: 04-X34-088 (08/06/2016).

## Author Contributions

L-LZ, E-PL, and H-PW conceived and designed the study. RK, SY, and S-CH collected and analyzed the data. L-LZ performed the statistical analysis. L-LZ and E-PL drafted the manuscript. H-PW designed and oversaw the study, interpreted the data, and revised the manuscript. All authors have read and approved the final manuscript for publication.

## Conflict of Interest

The authors declare that the research was conducted in the absence of any commercial or financial relationships that could be construed as a potential conflict of interest.
